# Characteristic analysis of adverse reactions of histone deacetylase inhibitors based on WHO-VigiAccess

**DOI:** 10.3389/fphar.2025.1563797

**Published:** 2025-03-18

**Authors:** Tongnan Yin, Yuyu Liu, Chenwen Li, Xinran Feng, Yumeng Lin, Zhongyu Qu

**Affiliations:** ^1^ Central Laboratory, Nanyang Central Hospital, Nanyang, China; ^2^ School of Integrative Medicine, Nanjing University of Chinese Medicine, Nanjing, China; ^3^ Department of Dermatology, Henan Provincial People’s Hospital, Henan University People’s Hospital, Zhengzhou, China; ^4^ Department of Preventive Dentistry, School of Stomatology, The Fourth Military Medical University, Xi’an, China; ^5^ Health Management Center, Nanjing Tongren Hospital, School of Medicine, Southeast University, Nanjing, China; ^6^ Department of Oncology, Nanyang Central Hospital, Nanyang, China

**Keywords:** histone deacetylase inhibitor, adverse drug reactions, chidamide, romidepsin, vorinostat, WHO-VigiAccess

## Abstract

**Background:**

This study assessed the adverse drug reactions (ADRs) associated with HDAC inhibitors using the VigiAccess database maintained by the World Health Organization (WHO). Furthermore, it compared the ADR profiles of three different drugs to identify the one with the lowest individualized risk for patients.

**Materials and methods:**

Data on adverse events of HDAC Inhibitors was retrieved from WHO-VigiAccess on 6 January 2025. We obtained data on age, gender, reporting year, and continent. Descriptive data related were calculated using Excel 2021. In this study, we used Excel software to analyze the characteristics of those who were harmed due to adverse reactions. For each drug, the reporting rate of adverse reactions was calculated by dividing the number of adverse reaction symptoms of this drug by the total number of adverse reaction reports. We listed the top 20 most frequent adverse reaction symptoms as common adverse reactions. By counting the frequency and proportion of these common adverse reactions, we conducted a comparative analysis of the adverse reaction situations of different drugs and classified them according to different types.

**Result:**

The WHO-VigiAccess database received 796, 1254, and 1658 ADR reports for Chidamide, Romidepsin, and Vorinostat respectively by 2024, with a total of 3,708. Gender distribution was relatively balanced (male:female ratio 0.81:1), and the 45–64 age group had the highest reporting rates, mostly from the Americas. Chidamide had higher rates in certain disorders, Romidepsin in others, and Vorinostat in specific ones. Common ADRs included thrombocytopenia etc., with some differences in rates among drugs. Serious ADR proportions were 0% for Chidamide, 2.27% for Romidepsin, and 1.02% for Vorinostat. 37 common signals were found, with Investigations having the most. Each drug had different ADR preferred terms (PTs) in renal/urinary and metabolism/nutrition disorders, with varying numbers of distinctive symptoms.

**Conclusion:**

Current comparative observational studies of these inhibitors indicate that there are both common and specific adverse reactions reported in the ADR data received by the WHO for these medications. Clinicians should enhance the rational use of these drugs by considering the characteristics of the reported ADRs.

## Introduction

Peripheral T-cell lymphoma (PTCL) is a group of highly heterogeneous malignant tumors that originate from mature T lymphocytes (Fiore et al., 2020). The proportion of PTCLs in all non-Hodgkin lymphomas is approximately 15%, and they display distinct clinical and biological features (Pizzi et al., 2018). PTCLs occur globally. The incidence rate in Europe and America accounts for 10%–15% of non-Hodgkin’s lymphomas, and it is even higher in Asia, about 20%–30%. There is no age limit for the onset of the disease, and the peak is at 60–70 years old. PTCLs mainly invade lymph nodes and are also prone to involve extranodal sites such as the skin, gastrointestinal tract, liver, spleen, and bone marrow. Patients often present with systemic symptoms such as fever, night sweats, and sudden weight loss, accompanied by fatigue, loss of appetite, and are prone to infections due to poor immunity (Pizzi et al., 2018). There are various pathological types of PTCL, however, the prognosis is relatively poor in all of them (Zing et al., 2018). Peripheral T-cell lymphoma, not otherwise specified (PTCL-NOS), is a relatively common subtype among peripheral T-cell lymphomas. The tumor cells have diverse morphologies and lack the characteristic manifestations of other specific subtypes, and the immunophenotype is usually positive for T-cell markers. Angioimmunoblastic T-cell lymphoma (AITL) has unique pathological features, including polymorphic tumor cells, accompanied by obvious vascular proliferation and increased immunoblasts, and often expresses markers such as CD10 and BCL-6. The tumor cells are usually large and have diverse morphologies, express CD30, and the ALK-positive subtype also expresses the ALK protein (Satou et al., 2022). Epigenetic modification does not change the DNA sequence but can regulate gene expression and alter cell functions and phenotypes. It is mainly achieved through DNA methylation, histone modification, and non-coding RNA regulation. They often co-occur and form a network to control the epigenetic system (Hara and Sawada, 2022). Histone acetylation and deacetylation are key regulatory methods of epigenetics and work synergistically in cell differentiation and development (Park and Kim, 2020). The imbalance of acetylation and deacetylation is closely related to many diseases including tumors (Gallinari et al., 2007; Horwitz, 2011).

HDAC inhibitors are closely related to the treatment of PTCLs (Horwitz, 2011). HDAC inhibitors, by inhibiting HDAC activity and restoring histone acetylation, enable tumor suppressor genes to function again. They can also regulate the tumor microenvironment, induce tumor cell cycle arrest, and improve the prognosis of patients (Zhang et al., 2019; Irimia and Piccaluga, 2024).

Chidamide, Romidepsin, and Vorinostat, acting as histone deacetylase inhibitors, were approved by FDA as novel antitumor agents. (Pojani and Barlocco, 2021; Li et al., 2019). These drugs prevent the proliferation of tumor cells by upregulating the cyclin-dependent kinase inhibitors p21 and p27, inhibiting the activity of CDKs, and causing cell cycle arrest in the G1 or G2/M phase. They promote tumor cell apoptosis by upregulating pro-apoptotic proteins such as Bax and Bak, downregulating anti-apoptotic proteins such as Bcl-2 and Bcl-xL, and activating the caspase cascade. They inhibit tumor growth and metastasis by suppressing the expression and secretion of angiogenesis-related factors such as VEGF and reducing tumor angiogenesis. They also enhance the immunogenicity of tumor cells by regulating the expression of immune-related molecules on the surface of tumor cells. Additionally, they regulate the expression of genes related to tumorigenesis and development by inhibiting the activity of histone deacetylases (Lu et al., 2023). This study evaluated the adverse drug reactions (ADRs) after using HDAC inhibitors in the VigiAccess database of the World Health Organization (WHO), and compared the ADR characteristics of three drugs to select the drug with the lowest individualized risk for patients.

## Materials and methods

Due to strict data protection laws and agreements between WHO PIDM members and the WHO, we are not be able to view individual case safety reports in VigiAccess. At the same time, VigiAccess divide the search results into groups both by active ingredient and continental region to avoid searches for specific brand names or individual WHO PIDM members. On 6 January 2025, we conducted a search through the WHO-VigiAccess platform for all adverse events reported following the use of the three HDAC inhibitors under investigation. We collected data on age, gender, reporting year, and related information from all continents. Following this, we performed descriptive statistical analyses on the data using Excel 2021.

WHO-VigiAccess is an integral component of the World Health Organization’s Global Pharmacovigilance Programme, functioning as a web-based drug safety database that aggregates data from global centers. The data is sourced from pharmacovigilance centers and related medical institutions in participating countries and regions worldwide. These entities collect, organize, and report data in accordance with established standards and norms to ensure accuracy and reliability. This platform primarily serves drug regulatory authorities, health professionals, research institutions, and pharmaceutical enterprises. Drug regulatory authorities utilize it to monitor the post-marketing safety of drugs and to formulate and adjust policies; health professionals access information to guide clinical medication; research institutions conduct relevant studies; and pharmaceutical enterprises gain insights into the safety of their products to enhance quality and risk management. MedDRA, or the Medical Dictionary for Regulatory Activities, was initiated for research and development by the International Council for Harmonisation of Technical Requirements for Pharmaceuticals for Human Use in 1996 and has since undergone continuous updates. It employs a five-level hierarchical structure: the top level is the System Organ Class (SOC), which categorizes medical concepts into 26 major system organ categories, such as the cardiovascular and respiratory systems; the second level is the High-Level Group Term (HLGT); the third level is the High-Level Term (HLT); the fourth level is the Preferred Term (PT), which describes specific medical events; and the bottom level is the Low-Level Term (LLT). MedDRA encompasses various fields, including disease diagnosis, symptoms, treatment measures, and adverse drug reactions, playing a crucial role in drug research and development, regulation, post-marketing monitoring, and medical research. It standardizes terminology, reduces ambiguity, promotes effective communication, ensures the safety of public medication, and supports drug risk management.

**FIGURE 1:**
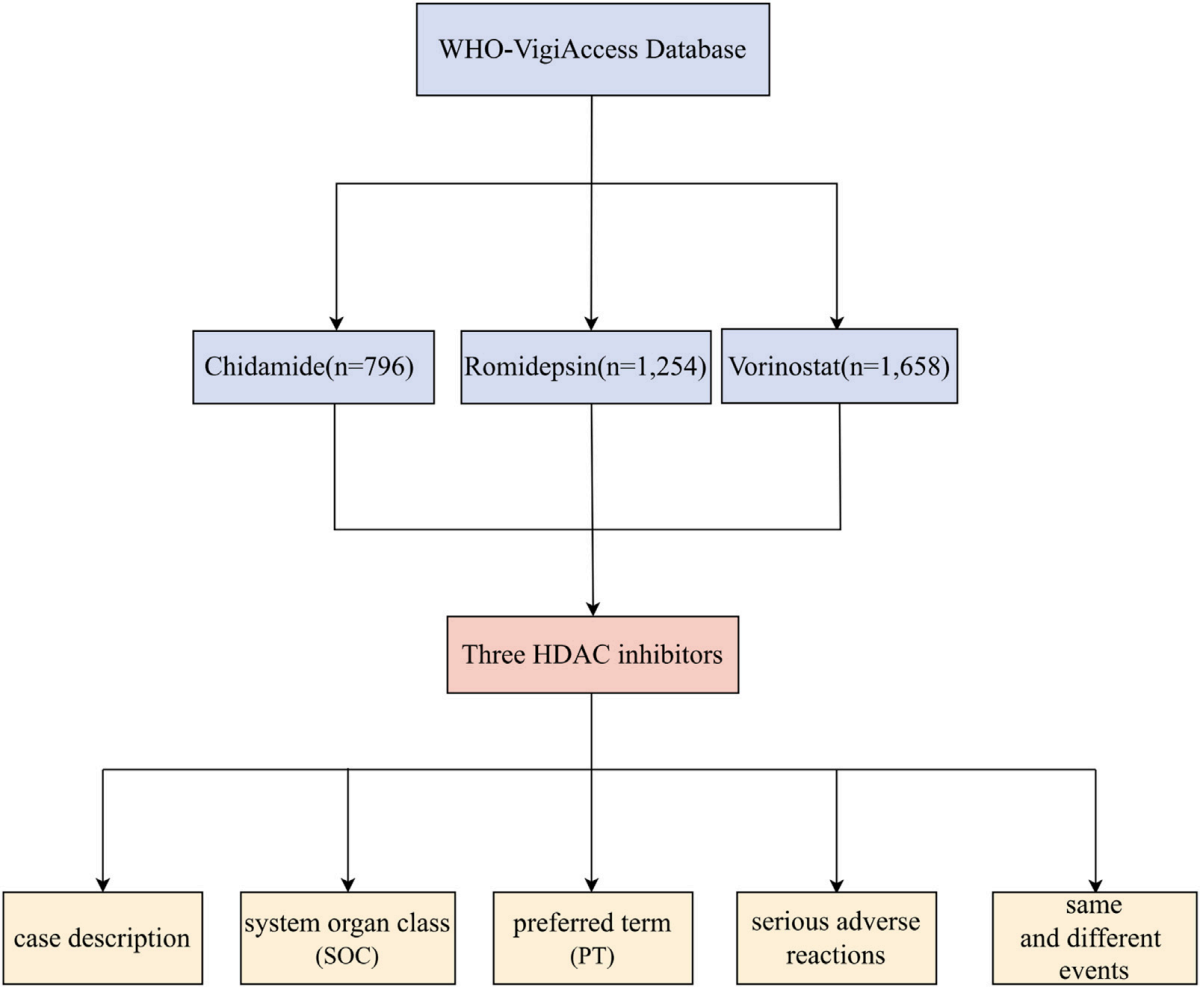
Flowchart for Adverse Reaction Analysis of three HDAC Inhibitors Based on WHO-VigiAccess Database.

In this study, we utilized Excel software to analyze the characteristics of individuals affected by adverse reactions. For each drug, the reporting rate of adverse reactions was calculated by dividing the number of adverse reaction symptoms associated with that drug by the total number of adverse reaction reports. We identified the top 20 most frequent adverse reaction symptoms as common adverse reactions. By assessing the frequency and proportion of these common adverse reactions, we conducted a comparative analysis of the adverse reaction profiles of different drugs and classified them according to various categories. Figure 1 shows the flowchart of adverse reaction analysis for three HDAC inhibitors.

## Results

### Case description of the study

The earliest reports of adverse reactions associated with the three HDAC inhibitors were received by WHO-VigiAccess in the following years: Chidamide in 2016, Romidepsin in 2009, and Vorinostat in 2006. As of 2024, WHO had received a total of 796, 1254, and 1658 ADR reports for these three drugs, resulting in a cumulative total of 3,708 reports. Our research conducted the following five - category analysis on these 3,708 adverse reports: case description, system organ class (SOC), preferred term (PT), serious adverse reactions, and same and different events. (Figure 1) Among the 3,708 reports related to these HDAC inhibitors, excluding 500 cases where gender was undetermined, the number of females experiencing ADRs (1,432 cases) was not significantly different from that of males (1,776 cases), yielding a male-to-female ratio of 0.81:1. In terms of age distribution, the group aged between 45 and 64 years exhibited the highest reporting rate, and 54.1% of the adverse reactions originated from the Americas. Additionally, Table 1 provides information on the reporting years for the studied drugs.

**TABLE 1:** Characteristics of ADR reports of three HDAC inhibitors.

Three HDAC inhibitors	Chidamide	Romidepsin	Vorinostat
Number of ADR reports	796	1254	1658
Female	355 (44.6%)	461 (36.8%)	616 (37.2%)
Male	429 (53.9%)	610 (48.6%)	737 (44.5%)
Unknown	12 (1.5%)	183 (14.6%)	305 (18.4%)
<18	11 (1.4%)	7 (0.6%)	167 (10.1%)
18–44	159 (20.0%)	96 (7.7%)	154 (9.3%)
45–64	261 (32.8%)	272 (21.7%)	442 (26.7%)
65–74	113 (14.2%)	264 (21.1%)	282 (17.0%)
>75	251 (31.5%)	192 (15.3%)	149 (9.0%)
Unknown	1 (0.1%)	423 (33.7%)	464 (28.0%)
Americas	1 (0.1%)	605 (48.2%)	1399 (84.4%)
Asia	795 (99.9%)	337 (26.9%)	162 (9.8%)
Europe		283 (22.6%)	82 (4.9%)
Oceania		29 (2.3%)	15 (0.9%)
Before2016	10 (1.3%)	326 (26.0%)	1245 (75.1%)
2017	171 (21.5%)	68 (5.4%)	54 (3.3%)
2018	122 (15.3%)	64 (5.1%)	61 (3.7%)
2019	266 (33.4%)	162 (12.9%)	55 (3.3%)
2020	139 (17.5%)	99 (7.9%)	49 (3.0%)
2021	17 (2.1%)	97 (7.7%)	48 (2.9%)
2022	24 (3.0%)	110 (8.8%)	70 (4.2%)
2023	25 (3.1%)	81 (6.5%)	53 (3.2%)
2024	22 (2.8%)	247 (19.7%)	23 (1.4%)

### Distribution of 20 system organ classes (SOCs) for three HDAC inhibitors

Table 2 presents the top 20 SOCs associated with the three HDAC inhibitor drugs. Notably, the reporting rates of Chidamide-related disorders in the Blood and lymphatic system, as well as General disorders and administration site conditions, and Investigations, were significantly higher compared to the other two HDAC inhibitors. Furthermore, Romidepsin exhibited significantly elevated rates of ADR reports related to Blood and lymphatic system disorders, Gastrointestinal disorders, General disorders and administration site conditions, Investigations, Infections and infestations, and Neoplasms, including benign, malignant, and unspecified types (such as cysts and polyps). In the case of Vorinostat, higher rates of ADRs were reported for Gastrointestinal disorders, Investigations, and General disorders and administration site conditions.

**TABLE 2:** ADR number and report rate of 20 SOCs of three HDAC inhibitors.

System organ classes	Chidamide (N = 796)	Romidepsin (N = 1254)	Vorinostat (N = 1658)
Blood and lymphatic system disorders	880 (110.55%)	406 (32.38%)	549 (33.11%)
Cardiac disorders	71 (8.92%)	123 (9.81%)	216 (13.03%)
Ear and labyrinth disorders	2 (0.25%)	8 (0.64%)	6 (0.36%)
Gastrointestinal disorders	471 (59.17%)	322 (25.68%)	742 (44.75%)
General disorders and administration site conditions	669 (84.05%)	517 (41.23%)	743 (44.81%)
Hepatobiliary disorders	11 (1.38%)	40 (3.19%)	71 (4.28%)
Immune system disorders		35 (2.79%)	27 (1.63%)
Infections and infestations	37 (4.65%)	230 (18.34%)	682 (41.13%)
Injury, poisoning and procedural complications	1 (0.13%)	128 (10.21%)	371 (22.38%)
Investigations	693 (87.06%)	459 (36.60%)	756 (45.60%)
Metabolism and nutrition disorders	17 (2.14%)	132 (10.53%)	461 (27.80%)
Musculoskeletal and connective tissue disorders	6 (0.75%)	35 (2.79%)	100 (6.03%)
Neoplasms benign, malignant and unspecified (incl cysts and polyps)		218 (17.38%)	238 (14.35%)
Nervous system disorders	3 (0.38%)	118 (9.41%)	363 (21.89%)
Psychiatric disorders	1 (0.13%)	23 (1.83%)	103 (6.21%)
Renal and urinary disorders	20 (2.51%)	37 (2.95%)	146 (8.81%)
Reproductive system and breast disorders	1 (0.13%)	9 (0.72%)	9 (0.54%)
Respiratory, thoracic and mediastinal disorders	12 (1.51%)	116 (9.25%)	417 (25.15%)
Skin and subcutaneous tissue disorders	23 (2.89%)	93 (7.42%)	162 (9.77%)
Vascular disorders	17 (2.14%)	43 (3.43%)	187 (11.28%)

### The most common adverse reactions for three HDAC inhibitors

Table 3 presents the preferred terms associated with the 20 most frequently reported SOCs for the three HDAC inhibitor drugs. All three HDAC inhibitors exhibited common ADRs, including Thrombocytopenia, Decreased Neutrophil Count, Decreased Platelet Count, Investigations, General Disorders and Administration Site Conditions, as well as Gastrointestinal Disorders. Notably, Chidamide demonstrated a significantly higher rate of ADR reports related to Decreased Neutrophil Count compared to the other two inhibitors. Additionally, Romidepsin had the highest rate of ADR reports for Decreased Platelet Count.

**TABLE 3:** Top20 ADRs of HDAC inhibitors.

Chidamide (N = 796)		Romidepsin (N = 1254)		Vorinostat (N = 1658)	
ADR	Report rate%	ADR	Report rate%	ADR	Report rate%
Thrombocytopenia	15.98	Platelet count decreased	3.97	Investigations	11.78
Neutrophil count decreased	14.58	Thrombocytopenia	3.75	General disorders and administration site conditions	11.58
Anaemia	11.14	Nausea	3.52	Gastrointestinal disorders	11.56
Asthenia	9.64	Death	2.27	Infections and infestations	10.63
Fatigue	9.30	Neutropenia	2.27	Blood and lymphatic system disorders	8.55
Nausea	6.07	Pyrexia	2.27	Metabolism and nutrition disorders	7.18
Vomiting	6.03	Fatigue	1.99	Respiratory, thoracic and mediastinal disorders	6.50
Pyrexia	3.54	Anaemia	1.95	Injury, poisoning and procedural complications	5.78
Diarrhoea	3.37	Vomiting	1.79	Nervous system disorders	5.66
White blood cell count decreased	2.52	Neutrophil count decreased	1.76	Neoplasms benign, malignant and unspecified (incl cysts and polyps)	3.71
Alanine aminotransferase increased	2.35	Disease progression	1.70	Cardiac disorders	3.37
Aspartate aminotransferase increased	1.91	Malignant neoplasm progression	1.54	Vascular disorders	2.91
Myelosuppression	1.74	Peripheral t-cell lymphoma unspecified	1.41	Skin and subcutaneous tissue disorders	2.52
Tachycardia	1.64	Asthenia	1.38	Renal and urinary disorders	2.27
Pneumonia	0.95	Diarrhoea	1.38	Psychiatric disorders	1.61
Blood creatinine increased	0.68	Decreased appetite	1.38	Musculoskeletal and connective tissue disorders	1.56
Electrocardiogram qt prolonged	0.61	White blood cell count decreased	1.18	Hepatobiliary disorders	1.11
Proteinuria	0.58	Product storage error	1.15	Surgical and medical procedures	0.51
Embolism	0.41	Atrial fibrillation	1.02	Immune system disorders	0.42
Cardiac failure	0.34	Febrile neutropenia	0.99	Eye disorders	0.28

### Three HDAC inhibitors associated with severe adverse events

**FIGURE 2:**
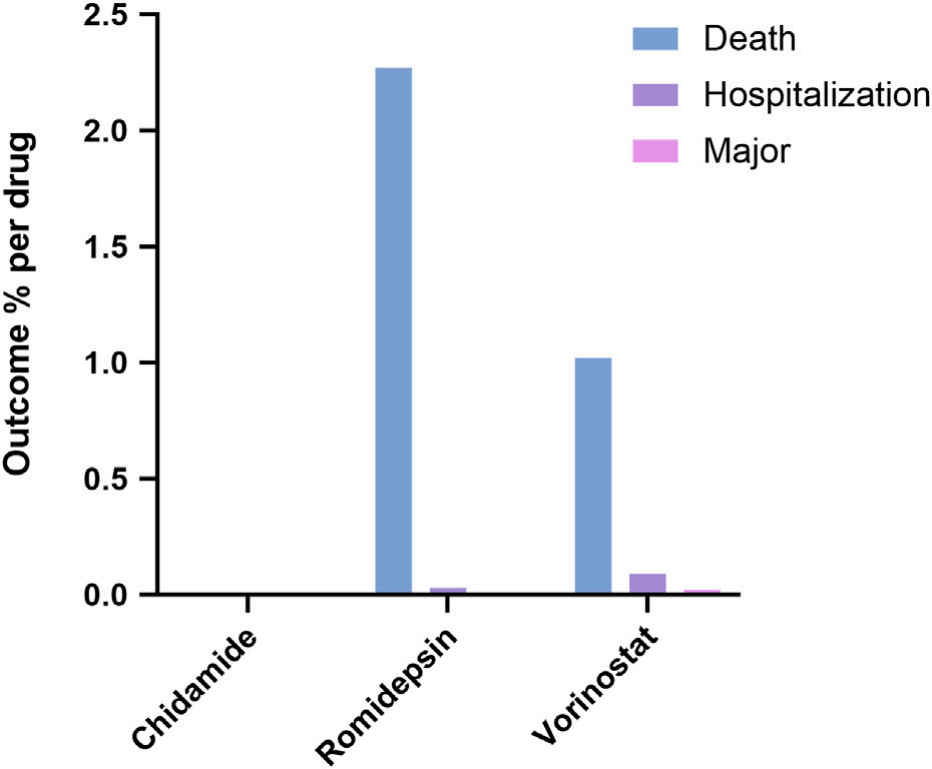
Outcomes for serious adverse events associated with three HDAC Inhibitors at the level of preferred terms.

In Figure 2, we identified the fatal adverse events associated with HDAC inhibitors using WHO-VigiAccess. The proportions of serious adverse reactions for the three inhibitors were as follows: Chidamide: 0%, Romidepsin: 2.27%, and Vorinostat: 1.02%.

### Same and different adverse reactions of three HDAC inhibitors

A total of 37 identical signals were identified at PTs for the three inhibitors by comparing the top 20 ADRs reported by each HDAC inhibitor within the SOCs. All common signals are classified in Table 4. The highest number of adverse signals was found in the SOC of Investigations, with the top five reports being Increased Blood Creatinine, Increased Blood Bilirubin, Prolonged Electrocardiogram QT, Decreased Haemoglobin, and Decreased Neutrophil Count. The Blood and Lymphatic System Disorders SOC ranked second, with the top five reports including Lymphopenia, Febrile Neutropenia, Anaemia, Myelosuppression, and Neutropenia. When comparing the top 20 ADRs reported by each HDAC inhibitor within the SOCs, it was observed that all three HDAC inhibitors exhibited different PTs of ADRs in the categories of Renal and Urinary Disorders, as well as Metabolism and Nutrition Disorders (see Table 5). Chidamide presented six distinctive symptoms, Romidepsin reported ninety-three, and Vorinostat identified one hundred and one, respectively.

**TABLE 4:** Same ADRs among three HDAC inhibitors.

System organ classes	ADRs	Signal N
Blood and lymphatic system disorders	Lymphopenia, Febrile neutropenia, Anaemia, Myelosuppression, Neutropenia, Pancytopenia, Thrombocytopenia	7
Cardiac disorders	Cardiac failure, Arrhythmia, Tachycardia	3
Gastrointestinal disorders	Nausea, Vomiting, Diarrhoea, Abdominal pain	4
General disorders and administration site conditions	Oedema, Pyrexia,Asthenia, Oedema peripheral, Fatigue	5
Hepatobiliary disorders	Hepatic function abnormal	1
Infections and infestations	Pneumonia	1
Investigations	Blood creatinine increased, Blood bilirubin increased, Electrocardiogram qt prolonged, Haemoglobin decreased, Neutrophil count decreased, Aspartate aminotransferase increased, Alanine aminotransferase increased, White blood cell count decreased	8
Metabolism and nutrition disorders	Hypokalaemia, Decreased appetite	2
Musculoskeletal and connective tissue disorders	Pain in extremity	1
Respiratory, thoracic and mediastinal disorders	Cough	1
Skin and subcutaneous tissue disorders	Pruritus, Rash	2
Vascular disorders	Embolism, Haemorrhage	2

**TABLE 5:** Different ADRs among three HDAC inhibitors.

System organ classes	Chidamide	Romidepsin	Vorinostat
Blood and lymphatic system disorders	Granulocytopenia, Erythropenia, Bicytopenia	Cytopenia, Platelet disorder, Disseminated intravascular coagulation, Haematotoxicity, Lymphocytosis	
Cardiac disorders		Bradyarrhythmia, Atrioventricular block second degree, Bundle branch block left, Cardiotoxicity, Cardiopulmonary failure, Myocarditis, Cardiac dysfunction, Cardiomyopathy, Cardiac tamponade	Sinus bradycardia, Left ventricular dysfunction, Myocardial is chaemia, Supraventricular tachycardia, Torsade de pointes, Atrial flutter, Cardiac arrest
Ear and labyrinth disorders		Ear pain, Deafness	
Gastrointestinal disorders		Swollen tongue, Pancreatitis acute, Flatulence, Mouth ulceration, Colitis is chaemic, Melaena, Lip swelling	Enterocolitis, Haematochezia, Rectal haemorrhage, Gastric haemorrhage, Proctalgia, Gastrooesophageal reflux disease, Intestinal perforation, Dysphagia, Haematemesis, Haemorrhoids, Colitis, Oesophagitis
General disorders and administration site conditions		Unevaluable event, Therapy partial responder, Injection site reaction, Infusion site extravasation, Injection site extravasation, Extravasation, Influenza like illness, Drug intolerance	Drug interaction, Adverse drug reaction,Non-cardiac chest pain, Feeling abnormal, Gait disturbance, Complication associated with device,No adverse event, Drug ineffective for unapproved indication
Hepatobiliary disorders		Hepatic cytolysis, Hepatic failure	Cholangitis, Hyperbilirubinaemia
Infections and infestations		Cytomegalovirus infection reactivation, Pneumonia *klebsiella*, Bronchitis, Urosepsis, *Clostridium difficile* colitis, Epstein-barr virus infection, Soft tissue infection, Nasopharyngitis, Conjunctivitis, Cytomegalovirus viraemia, Cytomegalovirus infection, Influenza, Pharyngitis, COVID-19, Hepatitis b, Epstein-barr virus infection reactivation	Clostridial infection, Herpes simplex, Diverticulitis, Hepatitis c,Upper respiratory tract infection, Appendicitis, *Escherichia* infection, Bacterial infection, *Escherichia* bacteraemia, *Clostridium difficile* infection, Enterococcal infection, Pneumonia fungal, Staphylococcal bacteraemia, Herpes zoster, Wound infection, *Pseudomonas* infection
Injury, poisoning and procedural complications		Product preparation issue, Intentional product use issue, Product label confusion, Product storage error, Product preparation error	Infusion related reaction, Product administered to patient of inappropriate age, Wrong technique in product usage process, Intentional product misuse, Head injury, Fracture, Underdose
Investigations		Laboratory test abnormal, C-reactive protein increased, Neutrophil count abnormal, Electrocardiogram st segment depression, Blood potassium increased, Blood potassium abnormal, Haemoglobin abnormal, Gamma-glutamyltransferase increased, Electrocardiogram abnormal, Oxygen saturation decreased, Liver function test abnormal, Platelet count abnormal	Blood sodium decreased, International normalised ratio increased, Activated partial thromboplastin time prolonged, Bacterial test positive, Haematocrit decreased, Fibrin d dimer increased, Blood pressure increased, Culture urine positive, Blood calcium decreased, Blood urea increased
Metabolism and nutrition disorders	Hypoproteinaemia, Appetite disorder	Hypomagnesaemia, Hypertriglyceridaemia, Metabolic acidosis	Acidosis, Diabetes mellitus, Hyperkalaemia, Failure to thrive, Hypervolaemia, Fluid intake reduced, Malnutrition
Musculoskeletal and connective tissue disorders		Rhabdomyolysis, Neck pain	Flank pain
Nervous system disorders		Peripheral sensory neuropathy, Cerebral haemorrhage, Sinus headache, Ageusia	Cerebral ischaemia, Nervous system disorder, Depressed level of consciousness, Memory impairment, Loss of consciousness, Aphasia, Hemiparesis, Paraesthesia, Balance disorder, Ataxia, Somnolence, Hydrocephalus, Cerebral infarction, Unresponsive to stimuli, Haemorrhage intracranial
Psychiatric disorders		Restlessness	Hallucination, Insomnia,Delirium, Disorientation,Agitation
Renal and urinary disorders	Proteinuria	Cystitis haemorrhagic, Urinary incontinence	Renal disorder, Haematuria,Renal impairment
Reproductive system and breast disorders		Amenorrhoea	
Respiratory, thoracic and mediastinal disorders		Organising pneumonia, Acute pulmonary oedema	Aspiration, Pulmonary haemorrhage, Haemoptysis, Respiratory distress
Skin and subcutaneous tissue disorders		Photosensitivity reaction, Skin irritation, Pyoderma gangrenosum, Rash macular, Urticaria, Stevens-johnson syndrome, Erythema multiforme, Rash erythematous, Dry skin	Dermatitis, Acute febrile neutrophilic dermatosis, Skin ulcer
Vascular disorders		Hot flush, Jugular vein thrombosis, Cyanosis	Thrombosis, Flushing

## Discussion

Epigenetic mechanisms are essential for the temporal and tissue-specific control of DNA transcription in various cell types (Pal and Tyler, 2016; Paluch et al., 2016). For instance, the acetylation of e-amino lysine residues of histones is an epigenetic modification. Histones package DNA in the cell nucleus. Thus, the degree of acetylation indirectly affects enzyme activity and strongly influences transcription.

In the occurrence and development of cancer, epigenetic alterations play a key role, among which DNA methylation and histone marking patterns are particularly prominent. DNA methylation promotes cancer progression; the imbalance of modifications such as acetylation and methylation of histones changes the chromatin state and affects gene expression. The epigenetic regulatory network composed of them, once disordered, will lead to abnormal gene expression and trigger cancer (Davalos and Esteller, 2023). Previous studies have shown that cancer metabolic remodeling has a profound impact on histone methylation and acetylation in the epigenome by altering the supply of intracellular metabolites. These epigenetic changes further regulate gene expression and promote the occurrence, development, and metastasis of cancer (Kinnaird et al., 2016). In the pathogenesis of Hodgkin lymphoma (HL), epigenetic changes are extremely crucial. It can restore the expression of tumor suppressor genes, inhibit the proliferation of tumor cells, etc.; it can also regulate immune-related genes, enhance the attack of the immune system on tumor cells, and improve the condition (Kirschbaum, 2011).

HDACs regulate the level of histone acetylation in cells and maintain a balance with acetylation under normal circumstances. However, in many types of cancers, HDACs are often overexpressed, leading to excessive histone deacetylation. This not only inhibits tumor suppressor genes but also indirectly activates oncogenes, promoting the proliferation of cancer cells and hindering their death. Previous studies have shown that HDAC inhibitors can inhibit the activity of HDACs, restore histone acetylation, induce apoptosis of cancer cells, and inhibit their proliferation (Bolden et al., 2006; Liu and Liou, 2023). In normal cells, histone acetylation and deacetylation maintain a dynamic balance to ensure normal physiological functions of the cells. HDAC inhibitors can specifically inhibit HDAC activity, prevent excessive histone deacetylation, restore its acetylation to the normal level, thereby loosening the chromatin structure and reactivating tumor suppressor genes (Carraway and Gore, 2007). The characteristics of most tumor cells are closely related to histone deacetylation. During tumor development, the activity of deacetylase such as HDAC abnormally increases, causing excessive histone deacetylation. HDAC inhibitors can inhibit the activity of deacetylase and to some extent curb the invasion and metastasis of tumor cells, becoming an effective strategy against tumors (Pratt, 2013). Previous studies have shown that the combination of HDAC inhibitors with other drugs for cancer treatment has more significant advantages than single treatment, providing a new idea for cancer treatment (Shah, 2019). Although HDAC inhibitors can be used in chemotherapy, their application is limited due to the related side effects caused by the weak selectivity for subtypes (Patel et al., 2022).

In our study, we analyzed the adverse reactions of three HDAC inhibitors - Chidamide, Romidepsin and Vorinostat by using the WHO-VigiAccess database. Before the study, we were already aware that only a few HDACi have received FDA approval, and most are currently undergoing clinical trials to determine their effectiveness in preventing and treating diseases (Shanmukha et al., 2023). Our selection of chidamide, romidepsin, and vorinostat was based on several considerations. Firstly, their prevalence in clinical practice and the availability of comprehensive data in the WHO-VigiAccess database. Secondly, by comparing drugs with diverse characteristics and approval statuses, we aimed to offer a broader perspective on the safety issues of HDAC inhibitors in PTCL treatment. Our findings revealed several important insights. Firstly, the gender distribution of adverse reactions was relatively balanced, with a slightly higher number of males reporting adverse events. The age group with the highest reported rates was predominantly between 45 and 64 years, suggesting that age may be a factor influencing the occurrence of adverse reactions. Geographically, the majority of reports originated from the Americas, which could be due to differences in drug usage patterns, reporting systems, or patient populations in different regions.

Adverse drug reactions may cause patients to develop new symptoms or worsen existing symptoms, affecting their physical health and quality of life. In severe cases, they may even endanger their lives, leading to serious consequences such as hospitalization, disability, or death.

Regarding the types of adverse reactions, each inhibitor exhibited distinct patterns. Chidamide was associated with higher rates of Blood and lymphatic system disorders, General disorders and administration site conditions, and Investigations. Romidepsin had elevated rates in Blood and lymphatic system disorders, Gastrointestinal disorders, and several other categories. Vorinostat showed a higher prevalence of Gastrointestinal disorders, Investigations, and General disorders and administration site conditions. Thrombocytopenia, Neutrophil count decreased, Platelet count decreased, Investigations, General disorders and administration site conditions, and Gastrointestinal disorders were common to all three. However, there were also notable differences in specific PTs within certain SOCs, particularly in Renal and urinary disorders and Metabolism and nutrition disorders. These differences emphasize the importance of individualized monitoring and management of patients treated with HDAC inhibitors. The proportion of serious adverse reactions varied among the three drugs, with Romidepsin having the highest rate at 2.27%, followed by Vorinostat at 1.02%, and Chidamide with 0%. These differences in serious adverse event rates further highlight the need for careful consideration of the risk-benefit profile when prescribing HDAC inhibitors.

Due to the differences in the types and severity of adverse reactions caused by different HDAC inhibitors, clinical doctors should choose appropriate drugs based on the patient’s specific condition (such as age, gender, medical history, comorbidities, etc.) when formulating treatment plans for patients. For example, the high incidence rate of Chidamide in blood and lymphatic system diseases, doctors should pay close attention to the relevant symptoms of patients in order to adjust the treatment plan in time. At the same time, especially for drugs with a high incidence of serious adverse reactions (such as Romidepsin), doctors should use them with caution and prepare measures to deal with possible serious adverse reactions, while fully informing patients of the relevant risks.

Although SRS has important value in monitoring adverse reactions, it is limited to few factors such as reputation bias, selection bias or under-reporting. From the current reports of AEs research results, the missing data cannot be classified as males, females or age groups. When we use databases such as WHO-VigiAccess, although adverse reactions can be mined, it is difficult to directly compare adverse reactions signals between different drugs due to the data accumulation and different time to market. This study compared the adverse reactions reporting rates of different drugs by collecting years of adverse reactions and PT data and minimized the impacts of drugs being marketed at different times as much as possible.

## Conclusion

This study utilized WHO-VigiAccess data to analyze the adverse reactions associated with three HDAC inhibitors: Chidamide, Romidepsin, and Vorinostat. Among these, Chidamide exhibited the lowest incidence of serious adverse reactions. The adverse reactions primarily affected the blood, lymphatic, and gastrointestinal systems. While HDAC inhibitors demonstrate efficacy against tumors such as T-cell lymphoma, they are also associated with significant side effects. Future research should aim to enhance subtype selectivity in order to mitigate these adverse reactions. Additionally, combination therapies may improve efficacy and address issues of drug resistance. Continuous monitoring is vital for optimizing treatment strategies.

## Data Availability

The original contributions presented in the study are included in the article/Supplementary Material, further inquiries can be directed to the corresponding authors.
